# Mr. Ring, on Vaccination

**Published:** 1806-09

**Authors:** John Ring


### *To the Editors of the Medical and Physical Journal.*

GENTLEMEN,

IN the last number of your Journal, I brought forward some proofs that the cow-pox originates from equine virus, contrary to the opinion of Dr. Kinglake. I now beg leave to add other instances, in which it seems to have arisen from the same cause. The first, which was communicated to me six years ago by Mr. Rankin, surgeon, of Eastbourne, was published in the year 1801, in the first volume of my Treatise on the Cow-pox.

Mr. Rankin was sent for to see a farmer, who had more than a dozen very large pocks, full of a semipellucid fluid, on his face and hands. They were elevated at the edges, and indented in the centre; and surrounded with a livid erysipelatous inflammation, and tumefaction. He had also a considerable degree of fever, a full quick pulse, violent headach, foul tongue, thirst, and much pain.

Mr. Rankin would not have been able to conjecture the cause of this disease, had not Captain Mowatt happened to shew him an account of Dr. Jenner's publication in a Review. On his next visit he inquired, whether any cattle on the farm had been affected in a similar way, and was informed, that a few days before the appearance of this disorder, the farmer had been dressing the heels of his horse for some scratches; and that some drops of a fluid from that part had spirted from the hairs of the heels into his face; which may be supposed to have had the greater effect on account of his having been just before shaved. This opinion was corroborated, by the circumstance of the pocks being particularly situated about the mouth and chin.

The sores were difficult to be healed; and the fever did not subside in less than a week. What renders it the more probable, that this farmer's complaint was occasioned by the cause here assigned, is, that his servant boy was affected in the same manner, though not so violently, and evidently from the same cause.

Mr. Tanner, the veterinary surgeon, of Rockhampton in Gloucestershire, applied some equine fluid to the teat of a cow, and produced the cow-pox; of which I have given an account in the publication before-mentioned; together with another case, seen by Mr. Henry Jenner, in which a boy, who first drew a string over the heels of a horse, and afterward afterwards across his fingers, caused an abrasion of the cuticle, and infected himself with the same disease. In the same work, I also mentioned three cases that had lately occurred, in which the cow-pox could be traced to the same source; one of them having fallen under the observation of Mr. Lawrence, surgeon, of Cirencester, and the others of Mr. Tanner.

In the same publication I ventured to express an opinion, that after the observations and experiments of Dr. Loy, in addition to others previously made, no doubt could remain of the origin of this distemper. In the beginning of the year 1801, Mr. Loy, surgeon, of Pickering, saw an eruptive disease on the hands of a farrier, who had been dressing the heels of a horse, similar to that described by Mr. Rankin. It consisted of distinct vesicles, regularly circumscribed; they had a dark speck in the centre, and were surrounded with an inflamed ring.

This case also is a sufficient proof, that equine virus is not inert, as Dr. Kinglake supposes; and it is an additional proof of its activity, that it produced such an effect in this man, notwithstanding he had previously had the small-pox.

The next case occurred in a man who had not had the small-pox. Vesicles appeared on both hands, particularly about the roots of the nails, while he was in the habit of dressing the heels of a horse affected with the grease. The lymphatics were inflamed as far as the armpit; where a swelling took place. A pustule, similar to those on the hands, was produced near the eyebrow; occasioned by his scratching that part, and inoculating it with his finger. He had also a considerable degree of fever.

With virus taken from this man, Mr. Loy excited in the human subject a similar disorder, and one exactly resembling the cow-pox. Dr. Tierney remarks, in his Inaugural Thesis, published at Glasgow, in April 1802, that if one understanding was so clouded, or one mind so perverse, as not to be convinced of the origin of the cow-pock, by the substantial evidence before offered to the public, not a shadow of doubt could remain in the mind of any person, after the experiments of Dr. Loy, who had published an account of them the preceding year, together with the observations of Mr. Loy, and his experiment as already related.

Apparently, my friend Dr. Tierney was too hasty in his conclusion. Apparently, there are understandings so clouded, and minds so perverse, as not to be convinced of of the origin of the disease, even by the evidence of Dr. Loy, together with the additional evidence that has been offered from different quarters, up to the present time. But having often conversed with those persons, whose understandings appeared to be so clouded, and whose minds appeared to be so perverse, I have uniformly found, that, like Dr. Kinglake, or the critics in Reviews, whatever was their learning in other respects, they were totally ignorant of every thing that had passed on the subject, except the unsuccessful experiments of Dr. Woodville, Mr. Simmons, and Mr. Coleman.

Dr. Loy inoculated the udder of a cow with the same virus; and produced a vesicle, surrounded with a margin, of a rose colour, attended with considerable induration and pain. The vesicle continued to increase for several days; and at length terminated in a scab. Two children were inoculated from this cow; and proved insusceptible of the small-pox.

Dr. Loy next inoculated another cow with limpid matter of grease; and the result was a vesicle, containing a watery fluid, of a purple tinge. A child was inoculated with this virus, and afterwards inoculated for the small-pox in three places, with the greatest care and attention; but resisted the infection of that disorder.

It is worthy of observation, that in this, and other instances related by Dr. Loy, so far from equine matter being inert, as supposed by Dr. Kinglake, the constitutional symptoms, though similar in kind, were more violent in degree, in their first and second remove from the horse. In five children, who were vaccinated from that, whose case was last mentioned, some trace of this virulence seemed to remain; but of the identity of its origin, there appears no reason to doubt, since they all resisted the infection of the small-pox.

Many of Dr. Loy's attempts to inoculate with the equine fluid proved unsuccessful. It is, however, proper to mention, that he inoculated three other cows with the same lymph that succeeded in the instance before mentioned; and excited in them all a similar disease. He is of opinion, that there are two species of grease; one local, and not infectious; the other infectious, preceded by fever and considerable indisposition, and relieved by a general eruption.

The following passage, in my publication before alluded to, is perhaps, alone, sufficient evidence on this head, to convince any rational man, that this is the origin of the cow-pock. "By a letter, dated Whitby, Dec. 26, 1802, Dr.

Dr. Loy informs Dr. Jenner, that the subject of his sixth experiment, who was inoculated with the matter of grease, has resisted the infection of the natural small-pox, although several times exposed. Several of those whom he had inoculated with vaccine virus, generated by inoculation with equine, have also been exposed more than once to the infection of the natural small-pox; but without effect.

"Dr. Duncan expressed a doubt, whether these persons had not had the small-pox previous to inoculation: but Dr. Loy affirms, that this supposition is perfectly groundless; and that the majority of them had never been within several miles of the small-pox, till after inoculation.

"That the variolous matter which he used was good, is evident from his having readily communicated the disease to others; and *many gentlemen of respectability in the neighbourhood can testify, that the experiments were fairly performed. One of them, at my request, saw me inoculate one of his cows from the greasy heel of his horse, with a lancet which he himself supplied me at the time of the experiment; and this experiment proved successful.*"

What can the most determined sceptic demand, or wish, more than this? How can folly itself expose itself more, than in expressing a doubt, or even a shadow of doubt, after this? The only way, in which I can account for the disbelief of any man in this case, is either to suppose that he is biassed by some preconceived notion; or that he has never read a single word on the subject.

It is true, men of the first celebrity have hesitated to believe this theory; but they were prejudiced by some previous representation of the case. Dr. Valentine, whom, when in England, Dr. Jenner referred to me for further information on this topic, confessed his doubt, and that of his countrymen, concerning it; but we must recollect, that they derived their early notions, as well as their early matter, from parties who were hostile to Dr. Jenner, and jealous of his fame.

Quo semel est imbuta recens, servabit odorem

Testa diu.

The many cases of coincidence between the occurrence of the grease in the horse, and the cow-pox, which have now come to the knowledge of Dr. Valentine, seem to have convinced him of the reality of its being the source of the disease. Accordingly, we find him adducing one remarkable case, in which it was transferred to the cow through the medium of another animal, and finally to the human subject; as I stated in your Journal for June, the very very number in which Dr. Kinglake expressed his doubt on this subject. If to this we add the recent case that follows in the same number, in which were at least five eye witnesses of the fact, namely, Dr. Jenner, Dr. Adams, Mr. Tanner, together with the farmer and his servant, Dr. Kinglake appears to be placed rather in an awkward situation; and, I humbly conceive, must either yield his assent to the controverted hypothesis, or pertinaciously adhere to his original tenet with an ill grace.

The next case, which is also recorded in my Treatise, is one that was communicated to me by Dr. Jenner, and to him by Lord St. Asaph; who informed him, that when he read his publication, this was the only point which he thought not sufficiently proved. His Lordship accordingly made some inquiries in the county of Suffolk, where he resides. The person to whom he applied, was Mr. Harwood, of Battisford Hall; who declared that he *positively disbelieved* the fact.

A few weeks after, he called on Lord St. Asaph; and told him, that he had very unexpectedly obtained some information on the subject. Samuel Nunn and his wife, who had been servants to Mr. Harwood, having lately visited him, Mrs. Nunn alluded to some event which occurred when all the cows had sore udders; and when she herself suffered so much pain from a violent eruption on her hands and arms, the marks of which were still visible.

On hearing this, and recollecting the questions put to him by Lord St. Asaph, Mr. Harwood asked the husband, if he remembered whether any horse in his stable had at that time greasy heels. He replied, that he certainly did; and named the particular horse that had been so diseased. It appeared, that it was not this man's business to milk the cows; but he was then paying his addresses to his wife, and, after finishing his own work, part of which was taking care of the horses, he sometimes went to assist her. He himself was not infected; but of twenty cows in the dairy, not a single one escaped.

"These facts," says his Lordship, "were communicated to me by an avowed disbeliever in the the truth of your discovery; and obtained from persons who had never heard of Dr. Jenner, or vaccine inoculation; one of whom indeed had had the disease, without knowing it, or its name. I flatter myself such ignorance, in a short time, will no more be found; and that, in this instance at least, we shall not 'see nations slowly wise,' in availing themselves of the invaluable advantages of the discovery; nor 'meanly just' to the merits of the man who made it."

The noble Lord was, however, too sanguine in his expectations. More than three years have elapsed since his letter was written; yet we still see nations, who are only 'slowly wise' in adopting Dr. Jenner's discovery, and only 'meanly just'—if at all just, in rewarding his merit.

In the same publication, I related other instances of successful inoculation with the matter of grease, by Dr. Sacco of Milan, and Dr. La Font of Salonica. The former affords the stronger evidence of the truth of this doctrine, on account of his having been at first sceptical on this point. He had also made a number of experiments, in order to decide the question, but in vain. At length, however, in consequence of a perusal of Dr. Loy's publication, he was induced to institute a new series of experiments; and the wetness of the season, which multiplied the sources of the virus, favoured his attempt.

His own servant, after dressing one of his horses labouring under that disorder, had five pustules on the forearms; but Dr. Sacco was not informed of the circumstance, till they were in a state of exsiccation. He tried the matter, but did not succeed.

This case, however, encouraged him to persevere in his efforts; which were at length crowned with success. A coachman, who had an eruption on his hands from the same cause, went to the Foundling Hospital at Milan; where several children were inoculated from him. On the same day Dr. Sacco also inoculated from him nine children and a cow. In the cow the infection failed; but in three of the children a pustule exactly similar to the cow-pock took place. From these Dr. Sacco repeated his experiments; and reproduced similar pustules to the fourth generation. He also submitted his patients to the test of variolous inoculation, which they bore with impunity; and hence he concludes, as every rational man, who is acquainted with the result of his experiments must conclude, that the grease is the origin of the cow-pock; and that equine and vaccine matter are the same.

These experiments were made in the presence of Dr. Malfatti of Vienna, and several other medical practitioners of Milan, Bologna, Pavia, and other parts of Italy, whom Dr. Sacco assembled on the occasion; all of whom concurred in opinion, that they afforded a complete confirmation of the theory of Dr. Jenner. He has, since that time, inoculated six other children with the matter of grease, grease, and excited two pustules, possessing all the characteristics of the cow-pock. He is therefore of opinion, that equine matter, when genuine, is a security against the small-pox, without passing through the medium of the cow.

In a former part of this Journal, I stated the contents of a letter from Dr. De Carro, of Vienna; in which he informed me, that he received some of this equine matter from Dr. Sacco; and that he was every day inserting equine and vaccine matter with perfect indifference, being fully convinced of their identity. I also communicated similar intelligence from Dr. Friese, the Director of Vaccination in Silesia, who received his supply of equine virus from Dr. De Carro, being part of the stock of Dr. Sacco. It is described as viscid, and of a darkish hue.

Dr. La Font, who also succeeded in inoculating with the matter of grease, is a French physician of great learning and ingenuity at Salonica; to whom Dr. De Carro sent Dr. Loy's publication, requesting him to make as many experiments as possible with equine matter. From him we learn, that the farriers in Salonica are well acquainted with the grease; and that they divide it into three distinct species, the phlegmonous, the scrofulous, and the variolous. With this last species, supposing it to be the same with that of Dr. Loy, Dr. La Font commenced his experiments.

The horse from which he took the virus had been attacked with symptoms of fever; which ceased as soon as the eruption appeared. His fore-legs were much swollen. On the left he had four pustules; one on the heel, a second some inches higher, a third on the articulation, and a fourth near the breast. They resembled the small-pox; but there was no eruption in any other part.

He took matter from the upper pustules on the twelfth day, which was limpid, but rather yellow and filamentous; and inoculated a cow, but without success. He next inoculated a girl twelve years of age; in whom also the experiment failed, but it is probable she had had the small-pox. He then inoculated two boys with the same matter; and in each of them produced an affection resembling the cow-pock; but attended with rather more fever. But in the subsequent inoculations, the disorder assumed its usual mild form.

As a farther confirmation, if any farther confirmation can be necessary, of the evidence of Dr. Sacco, I subjoined to his account the substance of a letter from a Me-

 dical dical Delegation of the Department of Ancona to Sir Benjamin West, President of the Royal Academy; stating, that Mr. Birago, a surgeon of Milan, had met with a similar case; and the Committee of Vaccination in that city had ascertained by experiments, that the virus contained in the vesicles produced an affection in the human subject, bearing a perfect resemblance to the cow-pock.

The case of ——— Perry, in St. Ann's Court, which I published in the same work, still farther corroborates the truth of this hypothesis. When he lived at Anstey, in Hertfordshire, he was employed in dressing horses and in milking cows; in consequence of which he had the guardian pock, which has left marks behind, and secured him from the small-pox; but it is uncertain, whether he received the infection of that disorder from the horse or the cow. It should also be recollected, that in the two instances where the genuine cow-pox appeared in the neighbourhood of London, soon after the promulgation of Dr. Jenner's discovery, it appeared in two great dairies, where men are alternately occupied in the care of horses, and in milking.

Many other instances of this kind might be mentioned; but no one is more remarkable than the following. John Elstone, of Mifflin, in Gloucestershire, caught a disease resembling the cow-pox, from the heels of a horse, in the year 1777. In the year 1801, he was inoculated with active vaccine virus in both arms, by Dr. Jenner, but to no purpose; although the operation succeeded in thirty other persons, who were inoculated with the same matter, at the same time. He was inoculated again, after the expiration of a fortnight; but in vain. No other reason can be assigned for his insusceptibility of the infection of the cow-pock, but his having contracted this disease from the horse; since he had never had the small-pox, nor milked a cow.

In addition to this accumulation of evidence on the positive side, I shall, in your next Journal, bring forward some very strong evidence on the negative side, similar to what has already been adduced on various occasions. It was lately received by the Royal Jennerian Society from Mr. Dalton, of Arnee; and is contained in a letter from him, published in the Madras Gazette, and dated 11th August, 1805, in answer to some observations on the same subject in the Madras Courier.

*August* 19, 1806. JOHN RING.

( To be continued. )

